# *In-operando* characterizations of oligothiophene OFETs: controlling the structure-property relationships at the nanoscale

**DOI:** 10.1186/s11671-025-04332-5

**Published:** 2025-08-16

**Authors:** Souren Grigorian, Anton Davydok, Linda Grodd, Yuriy Luponosov, Sergey Ponomarenko, Ilaria Fratoddi

**Affiliations:** 1 Department of Physics, University of Siegen, Walter-Flex-Strasse 3, 57068 Siegen, Germany; 2 Department of Chemistry, Sapienza University of Rome, P.le A. Moro 5, 00185 Rome, Italy; 3 Institute of Material Physics, Helmholtz Zentrum Hereon, Notkestr 85, 22607 Hamburg, Germany; 4 Enikolopov Institute of Synthetic Polymer Materials of Russian Academy of Sciences, Profsoyuznaya Str. 70, 117393, Moscow, Russian Federation

**Keywords:** Oligothiophenes, Ultrathin OFETs, α,α'-quinquethiophene (DH5T), Operando test, Synchrotron beam, GIWAXS

## Abstract

Grazing Incident Wide Angle X-ray Scattering (GIWAXS) studies on organic field-effect transistors (OFETs) fabricated with an aliphatic functionalized α,α'-quinquethiophene (i.e. 5,5′′′′-dihexyl-2,2′:5′,2′′:5′′,2′′′:5′′′,2′′′′-quinquethiophene, DH5T) thin film, were carried out. The structure-property relationships of the semiconductor material were investigated. A detailed, spatially resolved microstructural characterization of the active layer was carried out with the aim of understanding the role of the film’s microstructure on electrical performance. For this purpose, a custom-made setup designed for *in-operando* tests of OFETs was used, allowing a correlation under measured conditions of the complex microstructure with the thin film electrical behavior, under operating conditions. The GIWAXS measurements revealed a significant anisotropy of the DH5T thin films, under source-drain applied voltages (V_sd_). Particularly notable variations were observed for both in-plane and out-of-plane directions. Upon applying the V_sd_, the microstructure remained relatively stable in the out-of-plane (001) direction, suggesting that this orientation is less affected by the applied voltages. However, in the in-plane (020) direction, an increase of the π–π stacking of the DH5T molecules was found, indicating a stronger response of the microstructure to the applied voltage. Notably, a higher tensile strain, exceeding 1%, was observed at a V_sd_ of − 10 V, suggesting that the application of voltage induces significant structural reorganization in the thin film, which may have implications for optimizing the performance of OFETs in practical applications.

## Introduction

The intrinsic characteristics of organic single-crystal semiconductors make them exceptionally suited for a wide range of optoelectronic applications, including photovoltaics, and transistor applications, where they play a pivotal role in performance of electronic devices [1]. Their nearly flawless spatial arrangement and high crystallinity contribute to minimal defects, which in turn enhances electron mobility. These features position organic semiconductors as ideal candidates for high-performance transistors [2]. Recently these materials have emerged as promising candidates for wearable electronics, where flexibility and tunable electronic properties play a crucial role [3]. Small-molecule organic semiconductors exhibit pronounced π–π stacking features, which can be tuned by varying film preparation and applied stress [4]. Efficient electronic conjugation ensures high mobility of charge carriers between source and drain electrodes, thus facilitating rapid switching speeds in transistors [5].

Conjugated oligothiophenes and their derivatives have emerged as promising candidates for applications in organic electronics [6, 7]. It is noteworthy that their chemical stability ensures the long-term reliability in diverse operating conditions, making them suitable for the development of organic transistors [8]. The synthetic versatility of conjugated oligothiophenes allows for the precise control over their molecular structure, side-functionalities, molecular weight and, in turn, electronic properties. Moreover, the presence of aliphatic substituents in the main chin or as ending groups, enhances their solubility in common organic solvents [9]. This tunability is essential for tailoring transistors to meet specific application requirements [7], providing the versatility needed to attain desired electrical characteristics and endowing them for an extensive array of electronic devices, ranging from low-power sensors to high-speed processors, and various flexible electronic devices [10, 11]. Furthermore, a deep understanding of the lifespan and stability of organic semiconductors is necessary for ensuring sustained functionality and tuneable performances in electronic devices [12]. Among others, oligothiophenes with thiophene units in the 2–6 range have been extensively investigated for organic field-effect transistor (OFET) applications [13]. Specifically, 5,5′′′′-dihexyl-2,2′:5′,2′′:5′′,2′′′:5′′′,2′′′′-quinquethiophene (DH5T), a symmetrical alkyl-substituted quinquethiophene, stands out as a model compound of organic semiconductors [14].

Detailed characterization of OFET behaviour, including parameters such as conductivity, field-effect mobility, voltage sensitivity, and the applicable operating range, is essential for defining their practical use in devices [15]. Additionally, understanding the microstructure-property relationships in OFETs is also critical for optimizing their performances in optoelectronic applications [16]. Furthermore, the limited availability of high-quality, single organic crystals of appropriate sizes to match the channel dimensions of OFETs presents a challenge, requiring specific conditions for testing [17]. To address this, several methods are employed to study these materials, including in-operando impedance measurements [18] and interface-state distributions analysis in metal-insulating-semiconductor capacitors [19] as well as in-operando spectrometry [20]. Poly(3-hexylthiophene-2,5-diyl), (P3HT) a popular low band gap polymer, has been subject of extensive study using these techniques.

Among different, synchrotron characterization methods at the shallow incident angle such as grazing incidence wide-angle X-ray scattering (GIWAXS) or grazing incidence X-ray diffraction/scattering (GIXD/GIXS) have been established as effective tools for exploring these fundamental aspects in organic thin films and organic devices [21, 22]. Despite significant progress has been made in correlating the structure and device functionality of conjugated organic systems, a comprehensive understanding remains elusive. For instance, GIXD analysis of P3HT films revealed moderate structural changes in the out-of-plane (h00) series under linear current conditions up to 20 V, while a dramatic reduction in the structural order was observed in the non-linear current regime at higher voltages [23, 24]. Phase transitions in thin oligothiophene films have been explored through combined electrical measurements and in situ X-ray diffraction [25]. In another study, the correlations between ordering, phase transitions, and charge transport were investigated in thin α,α'-dihexyl-quarterthiophene films using combined structural and electrical in-situ investigations [26]. These studies underscore the need for an improved understanding of the relationship between microstructure and electronic properties to enhance the performance of organic electronic devices.

The impact of nanoscale structural changes on the material performances has been demonstrated in real-sized in-operando OFETs based on P3HT films, where spatially resolved structural changes were observed during operation [27, 28]. In addition to GIXD, other in-situ and in operando techniques have been applied to study these materials, such as X-ray absorption spectroscopy (XAS) and X-ray photoelectron spectroscopy (XPS), providing insights into the local chemical environment and electronic states during device operation [29, 30]. In-situ Raman spectroscopy has also been used to monitor molecular vibrations and charge transport dynamics under applied bias, shedding light on how the molecular packing and interactions evolve under different electrical stress [31]. Furthermore, in operando electron microscopy techniques, including scanning electron microscopy (SEM) and transmission electron microscopy (TEM), allow for real-time imaging of device morphology changes at the nanoscale during operation [32, 33]. Recently, we developed a new setup for in-operando probing of OFETs, which enables the observation of microstructural changes in real-time, revealing local-scale modifications within working organic transistors [34]. These advanced in-situ and in operando methods offer crucial insights into the dynamics of organic materials under real operating conditions and are essential for advancing the design and optimization of next-generation organic electronic devices.

In this paper spatially resolved GIWAXS studies combined with electrical measurements are presented. In operando studies have been carried out on DH5T oligothiophene thin films deposited on single channel OFETs. This approach provides a simultaneous structural and electrical characterization at the nanoscale of the local parameters that influence the microstructure and, in turn, the electrical performance of thin semiconducting films.

## Experimental part

### Sample preparation

The OFET testbeds (gold on silicon field-effect transistor substrates) with 20 devices on a single chip were purchased from Ossila Limited, Sheffield, United Kingdom. A Keithley 2612A dual-channel source meter was used to source and measure current at applied V_SD_ voltage in the range ± 10 V and fixed V_G_ of − 10 V. The 5,5′′′′-dihexyl-2,2′:5′,2′′:5′′,2′′′:5′′′,2′′′′-quinquethiophene (DH5T) oligothiophene [14] thin layer was thermally evaporated in a vacuum deposition chamber under high vacuum at 10^−6^ mbar with the evaporation rate of 0.2 Å/s at the 60 °C [35].The substrate temperature was controlled by thermocouple integrated in the system and thin film thickness of 89 nm was determination using X-ray reflectivity. A solution of DH5T in toluene with the concentration of 0.3 mg mL^−1^ was used for both *ex-situ* and in situ studies. The solution was stirred for half an hour at the temperature of around 50 °C. Silicon wafers with thermally grown 300 nm silicon dioxide were cut into plates of two different sizes: 10 × 10 mm^2^ and 18 × 18 mm^2^. Smaller substrates were used for *ex-situ* studies. Electric gold electrodes (around 80 nm thick) with a chromium adhesion layer of about 5 nm were thermally evaporated onto 18 × 18 mm^2^ substrates. Gold electrodes were evaporated with the channel length of 1 mm using a shadow mask.

### Synchrotron experiments

*In-operando* GIWAXS structural characterization of the DH5T oligothiophene thin films-based transistor was performed at Nanofocus Endstation of P03 beamline at PETRA III Synchrotron, DESY (Hamburg, Germany) [36]. The experiment was performed with monochromatic X-ray beam with photonic energy of 16.98 keV. X-ray beam was focused down to 300 nm^2^ by Kirkpatrick–Baez mirrors from JTEC. The measurements were performed in grazing geometry under shallow X-ray striking beam with controlled penetration depth. Scattered signal was recorded by CCD type Photonic Science area detector with pixel size of 62 × 62 µm^2^ and dimension of 2940 × 2940 pixels^2^ installed on distance 317 mm from a sample position. Two-dimensional scattering patterns were integrated into 1-dimensinal profiles with the use of software packages of DPDAK [37] and self-developed MATLAB routine.

## Results and discussion

A deep understanding of charge transport processes in organic semiconductors is of fundamental importance and examining the local structure under operating conditions shields light on their real time behaviour. In weakly scattering organic thin films, structural information is typically averaged across the entire film. Considering the OFET device architecture, the channel length is usually several orders of magnitude smaller than the width, typically ranging from a few microns to a few tens of microns. Therefore, sub-micron resolution is required to accurately characterize the microstructure across the conductive channels. To achieve this, nanobeam GIWAXS studies are combined with a multimeter to source and measure current and voltage on the oligothiophene thin film. This combined approach provides fundamental insight into the molecular-level structural features, the arrangement of organic chains, and the correlation with the electrical response of the conductive system.

**Fig. 1:**
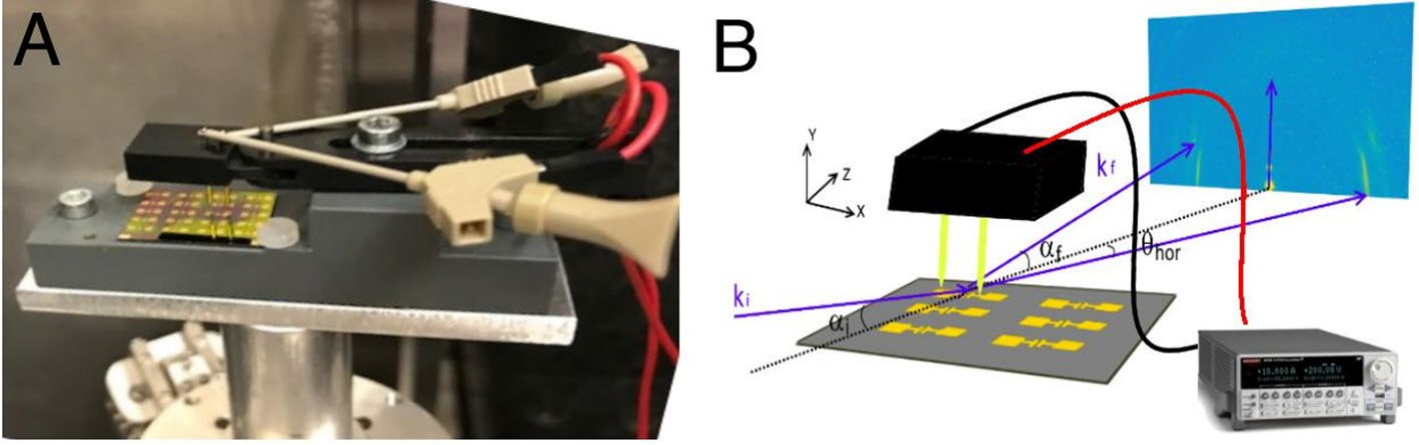
**A** OFETs probing setup and **B** schematic sketch of DH5T *in-operando* characterizations

Figure 1A illustrates the miniature setup used for *in-operando* GIWAXS characterization and local scale probing of DH5T-based OFET devices. The setup allows precise positioning of any transistors in the testbed. Source and drain electrodes are connected to Keithley digital multimeter via two adjustable gold electrodes. This configuration enabled to record the real-time current–voltage characteristics as schematically shown in Fig. 1B, during the *in-operando* characterization of the device.

The GIWAXS technique was used to observe changes in the microstructure of DH5T oligothiophene under externally applied voltages. By combining a synchrotron beam with an energy of 16.98 keV and large-area detector, the measures cover regions up to 3 Å^−1^ in reciprocal space in both directions in-plane and out-of-plane (i.e. parallel and perpendicular to the device surface). Due to the grazing incidence geometry with shallow incident angle, the beam footprint of 450 μm in direction along the striking beam covers entire transistor channel (Fig. 1B). In the perpendicular direction, the beam size is 300 nm providing high spatial resolution during lateral scans. All diffraction patterns were collected for 20 s per image, striking a balance between obtaining an intense signal and minimizing radiation damage. Spatially resolved scans through entire OFET channel were measured at initial stage, under voltage conditions and when voltage was released. GIWAXS measurements were performed at P03 Nanofocus Endstation, PETRA III (DESY, Hamburg).

**Fig. 2:**
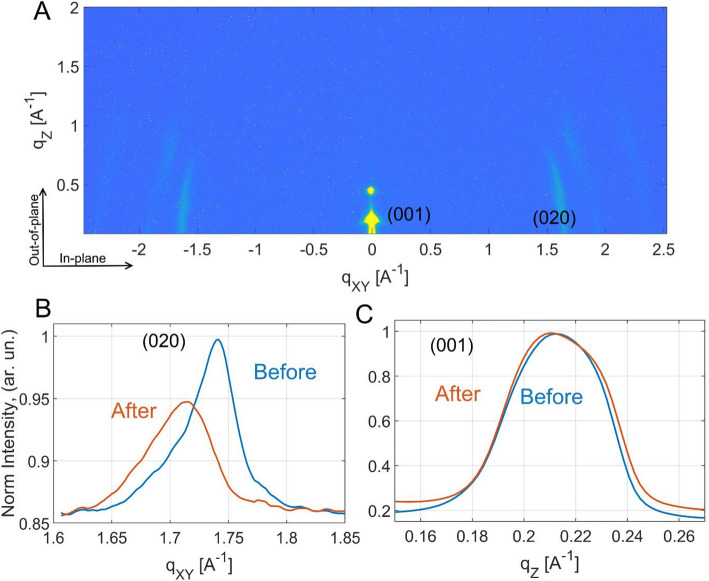
**A** The GIWAXS pattern of DH5T thin film; **B** the in-plane line profiles before (blue) and after (red) the working cycle; **C** the out-of-plane line profiles before (blue) and after (red) the working cycle

Experimental scattering pattern recorded at the initial stage of the *in-operando* experiment is shown in Fig. 2A. The pattern presented is recorded at the geometrical middle of OFET conductive channel at the incident angle of 0.045° degree.

The GIWAXS pattern shown in Fig. 2A corresponds to a monoclinic cell, where the molecules are found to be inclined with respect to a fully upright position along the π–π stacking direction [14]. The multilayer structure exhibited a surface morphology, which clearly reveals molecular monolayer terraces with an average thickness of 89 nm. For further analysis we have chosen two (001) and (020) peaks associated with long molecular axes and the π–π stacking. For both directions we compared the initial line profiles (blue) with the profiles (red) after the working cycle with − 2 V, − 5 V and − 10 V applied voltages (see Fig. 2B). The in-plane line profiles revealed substantial changes of the π–π stacking with expansion of the (020) interplanar distances and broadening of the Full width at half maximum (FWHM). At this point, it should be mentioned that the measurement of X-ray damage did not reveal any structural worsening due to X-ray irradiation. At the same time, the out-of-plane peak is stable after device operation without changes of the interplanar distances and marginal broadening of the peak (Fig. 2C).

**Fig. 3:**
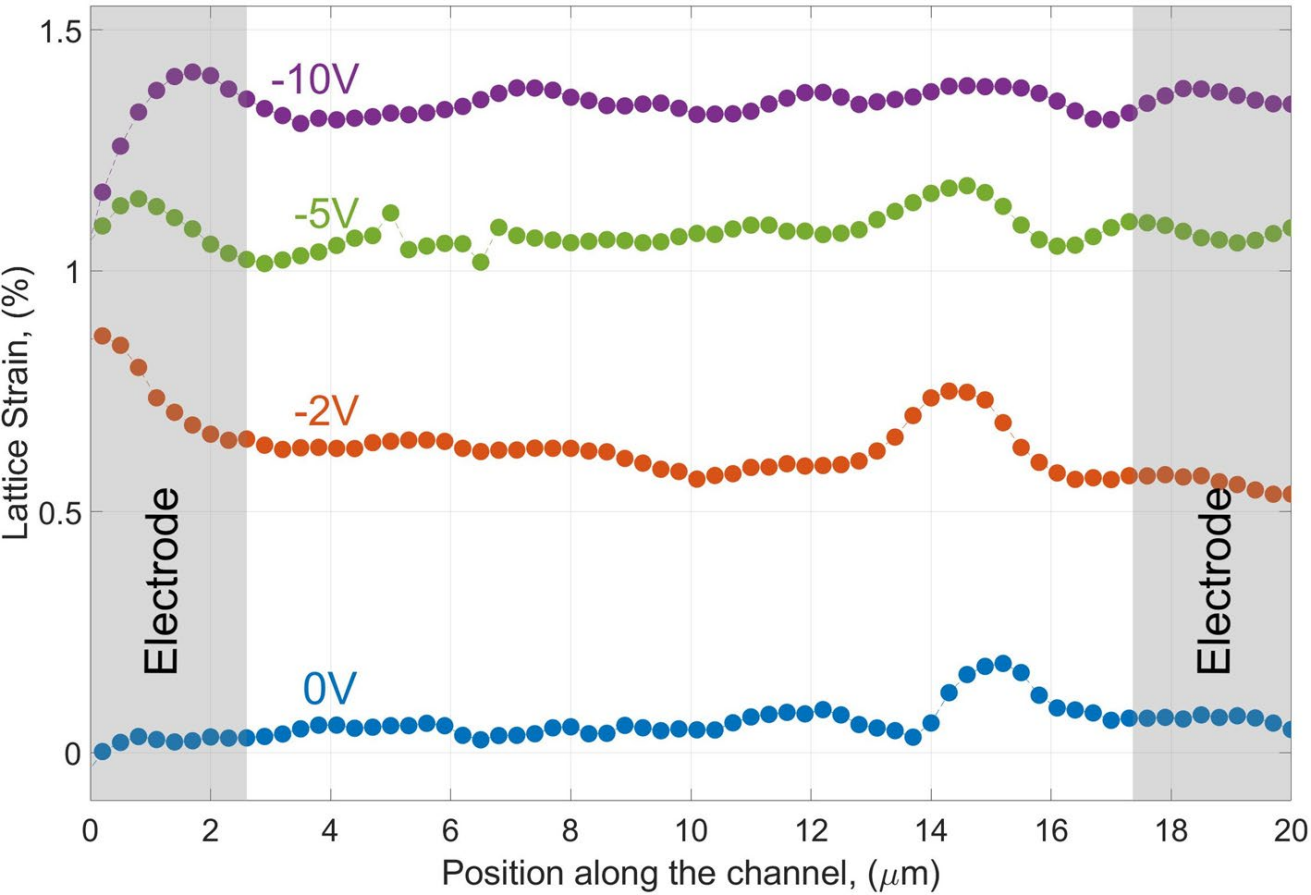
Lattice strain for in-plane profiles along the measured channel in a pristine state (blue curve) and under the applied voltages of − 2, − 5 and − 10 V_SD_ (red, green and magenta curves; respectively)

Further on, we analysed the π–π stacking behaviour with local resolution along the conducting channel. Starting and finishing at lateral positions on the gold electrodes we scanned within the steps size of 300 nm including whole conductive channel of 15 μm (see Fig. 3). When − 10 V voltage was applied, an average tensile strain of 1.3% was found. Spatially resolved measurements revealed a local variation of the tensile strain across the whole conductive channel shown in Fig. 3 as a function of the applied voltages. Interestingly, a local strain variation is observed along the channel, being present with up to 0.2% tensile strain even in the initial state before the device application (Fig. 3, blue curve). At the low voltages of − 2 V, this variation is still visible, however it's vanishing upon increasing of the applied voltage. The accuracy of the measurements is in the order of 10^–4^, indicating that the observed effect is real and represents residual strains, even at the initial stage. This highlights the advantage of using a nanobeam, as it enables the resolution of such small effects.

**Fig. 4:**
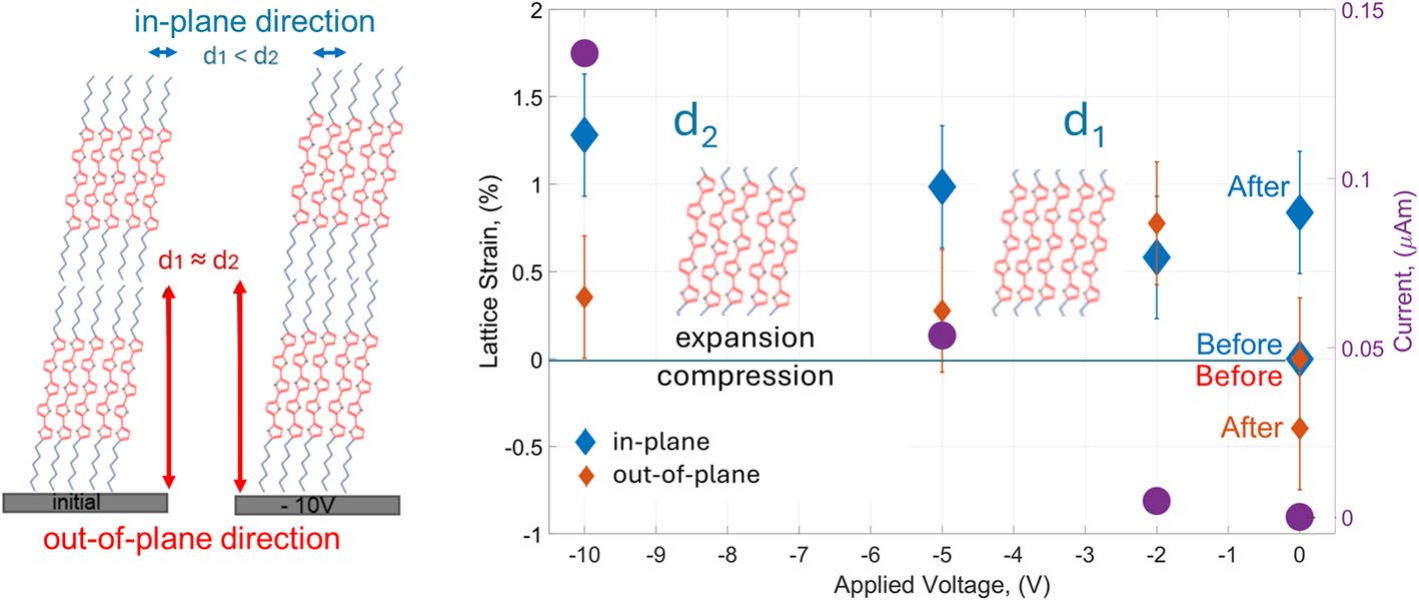
Left: Schematic representation of the initial DH5T arrangement and under a V_G_ and V_SD_ voltages of − 10 V; Right: Lattice strain provoked by externally applied V_SD_ voltage for in-plane (red) and out-of-plane (blue) directions before the voltage was applied, under − 2, − 5 and − 10 V and after the voltage was released

A more comprehensive understanding of the working device behaviour can be gleaned through an analysis of the average lattice strain observed at each voltage step, incorporating data from the electrodes and the channel with high-resolution precision. It is notable that the deviation of the d-spacing, as directly measured in the experiment, surpasses the values depicted solely for the channels in Fig. 3. These fluctuations are elucidated in Fig. 4, accompanied by corresponding error bars. Despite the apparent elevation of the lattice strain values, it is imperative to emphasize the precision achieved, measured in Angstrom units, which necessitates the utilization of nanoscale X-ray beams.

The sketch in Fig. 4, left schematically illustrated an initial arrangement of DH5T molecules and under the highest applied V_SD_ voltage of − 10 V for in-plane and out-of-plane directions. In Fig. 4, right both lattice directions are compared here (in-plane and out-of-plane) for the operating device at the different V_SD_ voltages and fixed V_G_ of − 10 V. Initially the system is unstrained with the position “Before” for both directions. Further on a clear tendency is observed: the higher level of the applied V_SD_ voltage coincides well with the expansion of in-plane π–π stacking. The lattice behaviour at the given V_SD_ value can be directly correlated with of current–voltage characteristics of the OFET (magenta filled circles).

In general, a higher stability in the out-of-plane direction with minor changes was found at all the applied voltages and after the device operation. From another point of view, the π–π stacking in the in-plane direction is proved to be more sensitive to applied voltages with a monotonous increase of the tensile strain and the maximal strain of 1.3% at − 10 V. These changes are partially irreversible after a device cycle with remaining tensile strain of about 1% for π–π stacking. It should be noted here that the reduction of the applied voltages favours better performance of the OFET devices considering that significant progress has been made in lowering the operation power consumption of the OFETs [38].

Devices based on organic semiconductors thin films have been widely studied to investigate the role of molecular packing, grain boundaries, defects and morphology on the charge transport phenomena [39]. For instance, the structural evolution of pentacene thin films was studied during device operation, highlighting the correlation under measured conditions of molecular reorientation with strain upon bias stress [40]. In our case, the partial irreversibility of the in-plane π–π stacking strain—evidenced by a residual tensile strain of approximately 1% after the voltage was released—suggests that structural fatigue may occur in the active layer during device operation. Such residual strain, while not directly quantified in terms of electrical performance in this study, may contribute to long-term device instability, including threshold voltage drift, hysteresis, or reduced mobility. This is consistent with previous reports that associate accumulated mechanical deformation in organic semiconductors with the formation of charge traps and mobility degradation. While our current data do not directly measure hysteresis or performance decay, these results underline the importance of minimizing mechanical strain—through materials design or voltage scaling—to enhance device lifetime. Whitin the limits of the present study, i.e. the choice of a single molecule, the voltage range, or spatial resolution constraints, these measurements allow us to observe the structural features with a resolution up to a few hundred nm. A particular advantage of the proposed approach is direct probing of the local scale structural changes and correlating with the working device performances. This approach is fundamental as a detailed simultaneous structural and electrical characterization of thin films at the molecular level can also be performed on the molecules with different backbone or end-cap substitutions.

## Conclusions

The *in-operando* approach presented in this work enables detailed microstructural characterization of the α,α'-dihexyl-quinquethiophene based OFETs, providing a correlation between the local structure of the conjugated oligomer and the device performance. A strong directional anisotropy in both in-plane and out-of-plane directions of the working OFET was observed. The out-of-plane direction remained nearly stable during the device operation by varying applied voltages. In contrast, the π–π stacking in the in-plane direction expanded under the applied voltages. The higher tensile strain of about 1.3% was observed upon applying − 10 V. Afterwards, releasing the applied voltage, partially reversible microstructural changes were observed. These findings are significant, as the detailed microstructural and simultaneous electrical characterization of thin films at the molecular level can be applied to different molecular backbone or end-substitutions. A comprehensive study of the microstructural parameters that influence the stability of organic semiconductor materials, along with a detailed analysis of their relationship to electrical performance, is critical for advancing the understanding of material behaviour. This integrated approach allows researchers to identify the key drivers of functionality and performance. By establishing these key links, it becomes possible to develop a systematic and strategic framework for designing advanced organic semiconductor materials with improved performance. Such progress is poised to drive significant breakthroughs in technologies like flexible electronics, photovoltaics, and next-generation transistors, accelerating innovation in the field of organic electronics. In summary, *in-operando* characterization demonstrated an important role in revealing the structure-property relationships of semiconducting oligomer thin films, enabling the direct observation of changes during device operation. These findings have important implications for the future design and optimization of organic field-effect transistors. By enabling real-time, spatially resolved observation of microstructural changes under operational conditions, our operando methodology provides a powerful diagnostic tool for identifying structural features that influence charge transport and device stability. This capability can inform the selection of molecular architectures and processing conditions that minimize strain-induced degradation, enhance charge mobility, and reduce operating voltages. Ultimately, such insights are critical for guiding the development of more reliable, efficient, and scalable organic electronic devices for applications in flexible electronics, sensors, and low-power circuits.

## Data Availability

Data sets generated during the current study are available from the corresponding author on reasonable request.

## References

[1] Zhang X, Dong H, Hu W. Organic semiconductor single crystals for electronics and photonics. Adv Mater. 2018;30: 1801048. 10.1002/adma.201801048. 10.1002/adma.201801048 30039629

[2] Duan S, Geng B, Zhang X, Ren X, Hu W. Solution-processed crystalline organic integrated circuits. Matter. 2022;4:3415–43. 10.1016/j.matt.2021.09.002.

[3] Liao C, Xiong Y, Fu Y, Chen X, Occhipinti LG. Organic semiconductors based wearable bioelectronics. Wearable Electr. 2025;2:23–39. 10.1016/j.wees.2024.12.003.

[4] Jun B, Lee CH, Lee SU. Strain-induced carrier mobility modulation in organic semiconductors. J Ind Eng Chem. 2022;107:137–44. 10.1016/j.jiec.2021.11.042.

[5] Massetti M, Zhang S, Harikesh PC, Burtscher B, Diacci C, Simon DT, Liu X, Fahlman M, Tu D, Berggren M, Fabiano S. Fully 3D-printed organic electrochemical transistors. NPJ Flex Electron. 2023;7: 11. 10.1038/s41528-023-00245-4.

[6] Uhrich C, Schueppel R, Petrich A, Pfeiffer M, Leo K, Brier E, Kilickiran P, Baeuerle P. Organic thin-film photovoltaic cells based on oligothiophenes with reduced bandgap. Adv Funct Mater. 2007;17:2991. 10.1002/adfm.200600917.

[7] Zhang C, Zhu X. N-type quinoidal oligothiophene-based semiconductors for thin-film transistors and thermoelectrics. Adv Funct Mater. 2020;30: 2000765. 10.1002/adfm.202000765.

[8] dos Santos MC, Pickholz M. The electronic structure of oligothiophenes. J Non-Cryst Solids. 2004;338:586–9. 10.1016/j.jnoncrysol.2004.04.001.

[9] Facchetti A, Yoon MH, Stern CL, Hutchison GR, Ratner MA, Marks TJ. Building blocks for N-type molecular and polymeric electronics. perfluoroalkyl- versus alkyl-functionalized oligothiophenes (nts;n)2–6). systematic synthesis spectroscopy electrochemistry and solid-state organization. J Am Chem Soc. 2004;126:13480–501. 10.1021/ja048988. 15479105 10.1021/ja048988a

[10] Fuller CW, Padayatti PS, Abderrahim H, Merriman B. Molecular electronics sensors on a scalable semiconductor chip: a platform for single-molecule measurement of binding kinetics and enzyme activity. PNAS. 2022;119: e2112812119. 10.1073/pnas.211281211. 35074874 10.1073/pnas.2112812119 PMC8812571

[11] Liu K, Ouyang B, Guo X, Guo Y, Liu Y. Advances in flexible organic field-effect transistors and their applications for flexible electronics. Flexible Electr. 2022;6:1. 10.1038/s41528-022-00133-3.

[12] Schaack C, Evans AM, Ng F, Steigerwald ML, Nuckolls C. High-performance organic electronic materials by contorting perylene diimides. J Am Chem Soc. 2022;144(1):42–51. 10.1021/jacs.1c11544. 34937338 10.1021/jacs.1c11544

[13] Zhang L, Colella NS, Cherniawski BP, Mannsfeld SCB, Briseno AL. Oligothiophene semiconductors: synthesis, characterization, and applications for organic devices. ACS Appl Mater Interfaces. 2014;6:5327–43. 10.1021/am4060468. 24641239 10.1021/am4060468

[14] Mikayelyan E, Bakirov AV, Shcherbina MA, Chvalun SN, Ponomarenko SA, Grigorian S. Real time studies of thiophene-based conjugated oligomer solidification. RSC Adv. 2015;5:1319–22. 10.1039/C4RA13109F.

[15] Larik FA, Faisal M, Saeed A, Abbas Q, Kazi MA, Thebo AA, Khan DM, Channar PA. Thiophene-based molecular and polymeric semiconductors for organic field effect transistors and organic thin film transistors. J Mater Sci Mater Electron. 2018;29:17975–8010. 10.1007/s10854-018-9936-9.

[16] Kukhta NA, Luscombe CK. Gaining control over conjugated polymer morphology to improve the performance of organic electronics. Chem Commun. 2022;58:6982–97. 10.1039/D2CC01430K. 10.1039/d2cc01430k 35604084

[17] Hao Z, Wu Z, Liu S, Tang X, Chen J, Liu X. High-performance organic thin-film transistors: principles and strategies. J Mater Chem C. 2024;12:9427. 10.1039/D4TC01240B.

[18] Wu R, Matta M, Paulsen BD, Rivnay J. Operando characterization of organic mixed ionic/electronic conducting materials. Chem Rev. 2022;122(4):4493–551. 10.1021/acs.chemrev.1c00597. 35026108 10.1021/acs.chemrev.1c00597

[19] Hatta H, Miyagawa Y, Nagase T, Kobayash T, Hamada T, Murakami S, Matsukawa K, Naito H. Determination of interface-state distributions in polymer-based metal-insulator-semiconductor capacitors by impedance spectroscopy. Appl Sci. 2018;8(9):1493. 10.3390/app8091493.

[20] Nagamura N, Kitada Y, Tsurumi J, Matsui H, Horiba K, Honma I, Takeya J, Oshima M. Chemical potential shift in organic field-effect transistors identified by soft X-ray operando nano-spectroscopy. Appl Phys Lett. 2015;106: 251604. 10.1063/1.4922902.

[21] Rivnay J, Mannsfeld SCB, Miller CE, Salleo A, Toney MF. Quantitative determination of organic semiconductor microstructure from the molecular to device scale. Chem Rev. 2012;112:5488. 10.1021/cr3001109. 22877516 10.1021/cr3001109

[22] Hexemer A, Müller-Buschbaum P. Advanced grazing-incidence techniques for modern soft-matter materials analysis. IUCrJ. 2015;2:106. 10.1107/S2052252514024178. 25610632 10.1107/S2052252514024178 PMC4285885

[23] Grigorian S, Tranchida D, Ksenzov D, Schäfers F, Schönherr H, Pietsch U. Structural and morphological changes of P3HT films in the planar geometry of an OFET device under an applied electric field. Eur Polym J. 2011;47(12):2189. 10.1016/j.eurpolymj.2011.09.003.

[24] Sajjad M, Liu X, Zhang F. Structural evolution of poly(3-hexylthiophene) thin films under electrical bias: A GIXD study. J Mat Sci. 2017;52:10389–98. 10.1007/s10853-017-0912-4.

[25] Mikayelyan E, Grodd L, Ksianzou V, Wesner D, Rodygin AI, Schönherr H, Luponosov YN, Ponomarenko SA, Ivanov DA, Pietsch U, Grigorian S. Phase transitions and formation of a monolayer-type structure in thin oligothiophene films: exploration with a combined in situ x-ray diffraction and electrical measurements. Nanoscale Res Lett. 2019;14(1):185. 10.1186/s11671-019-3009-8. 31147864 10.1186/s11671-019-3009-8 PMC6542962

[26] Liu Y, Zhang C, Wang T. In-situ study of charge transport and phase transitions in α,α’-dihexyl-quarterthiophene films. Org Electron. 2019;68:49–57. 10.1016/j.orgel.2019.01.020.

[27] Grodd LS, Mikayelyan E, Dane T, Pietsch U, Grigorian S. Local scale structural changes of working OFET devices. Nanoscale. 2020;12:2434. 10.1039/C9NR07905J. 31746902 10.1039/c9nr07905j

[28] Smith D, Johnson M, Lee J. Real-time structural monitoring of P3HT organic field-effect transistors during operation. Adv Func Mater. 2020;30:1907881. 10.1002/adfm.201907881.

[29] Huang L, Li J, Xu J. In-situ X-ray absorption spectroscopy of organic semiconductors under operational conditions. J Phys Chem C. 2018;122:8189–96. 10.1021/acs.jpcc.8b03112.

[30] Jiang S, Dai Q, Guo J, Li Y. In-situ/operando characterization techniques for organic semiconductors and devices. J Semicond. 2021;43: 041101. 10.1088/1674-4926/43/4/041101.

[31] Zhang H, Li Z, Chen Y. In-situ raman spectroscopy for monitoring charge transport in organic semiconductors. J Raman Spectrosc. 2016;47:743–9. 10.1002/jrs.4902.

[32] Jones A, Thompson K, Wang Z. Operando SEM imaging of morphological changes in organic thin-film transistors. Nano Lett. 2018;18:3062–8. 10.1021/acs.nanolett.8b01467.

[33] Park J, Lee S. Real-time TEM observation of morphology evolution in operating organic transistors. Nat Commun. 2020;11:2347. 10.1038/s41467-020-16377-w.32376830

[34] Davydok A, Luponosov Y, Ponomarenko SA, Grigorian S. In situ coupling applied voltage and synchrotron radiation: operando characterization of transistors. Nanoscale Res Lett. 2022;17: 22. 10.1186/s11671-022-03662-y. 35107638 10.1186/s11671-022-03662-y PMC8811105

[35] Mikayelyan E, Vladimirov I, Wesner D, Grodd L, Rodygin AI, Schönherr H, Ponomarenko SA, Pietsch U, Ivanov DA, Grigorian S. Impact of substrate temperature on the structure and electrical performance of vacuum-deposited α,α′-DH5T oligothiophene thin films. RSC Adv. 2016;6:115085–91. 10.1039/C6RA24609E.

[36] Krywka C, Keckes J, Storm S, Buffet A, Roth SV, Döhrmann R, Müller M. Nanodiffraction at MINAXS (P03) beamline of PETRA III. J Phys Conf Ser. 2013;425:072021. 10.1088/1742-6596/425/7/072021.

[37] Benecke G, Wagermaier W, Li C, Schwartzkopf M, Flucke G, Hoerth R, Zizak I, Burghammer M, Metwalli E, Müller-Buschbaum P, Trebbin M, Förster S, Paris O, Roth SV, Fratzl P. A customizable software for fast reduction and analysis of large X-ray scattering data sets: applications of the new DPDAK package to small-angle X-ray scattering and grazing-incidence small-angle X-ray scattering. J Appl Cryst. 2014;47:1797–803. 10.1107/S1600576714019773. 25294982 10.1107/S1600576714019773 PMC4180741

[38] Ren X, Lu Z, Zhang X, Grigorian S, Deng W, Jie J. Low-voltage organic field-effect transistors: challenges, progress, and prospects. Mater Lett. 2022;4(8):1531. 10.1021/acsmaterialslett.2c00440.

[39] Liscio F, Albonetti C, Broch K, Shehu A, Quiroga SD, Ferlauto L, Frank C, Kowarik S, Nervo R, Gerlach A, Milita S, Schreiber F, Biscarini F. Molecular reorganization in organic field-effect transistors and its effect on two-dimensional charge transport pathways. ACS Nano. 2013;7(2):1257–64. 10.1021/nn304733w. 23350706 10.1021/nn304733w

[40] Liscio F, Ferlauto L, Matta M, Pfattner R, Murgia M, Rovira C, Mas-Torrent M, Zerbetto F, Milita S, Biscarini F. Changes of the molecular structure in organic thin film transistors during operation. J Phys Chem C. 2015;119(28):15912–8. 10.1021/acs.jpcc.5b03901.

